# Perioperative Outcomes, Comorbidities, and Complications following Total Shoulder Arthroplasty in Wheelchair Users: A Retrospective Cohort Analysis of a Nationwide Database

**DOI:** 10.3390/jcm12185799

**Published:** 2023-09-06

**Authors:** Kevin Prabhu, Andrew J. Nasr, Donald Kasitinon, Alison Cabrera, Yen-Sheng Lin

**Affiliations:** 1 UT Southwestern Medical Center, Medical School, Dallas, TX 75390, USA; 2 Department of Applied Clinical Research, University of Texas Southwestern, Dallas, TX 75390, USA; 3 Department of Physical Medicine and Rehabilitation, University of Texas Southwestern, Dallas, TX 75390, USA; 4 Department of Orthopaedic Surgery, University of Texas Southwestern, Dallas, TX 75390, USA

**Keywords:** total shoulder arthroplasty, wheelchair, PearlDiver database, revision, complications

## Abstract

Impaired shoulder function hinders the ability of wheelchair users to maintain independence. The current state of the literature delineates the risks and benefits of surgical techniques for the management of shoulder pathologies. To the best of our knowledge, there is no study that has investigated complications following total shoulder arthroplasty (TSA) in wheelchair users. Utilizing the PearlDiver Mariner national administrative database, 72,108 patients were identified who underwent TSA with a concurrent diagnosis of a rotator cuff tear. Two matched cohorts, one of wheelchair users and one of non-wheelchair users, were created. Due to limitations within PearlDiver, one-year outcomes, including comorbidity and complication rates and readmission statistics, were compared between the two cohorts. Each matched cohort of 869 patients underwent TSA with a concurrent diagnosis of a rotator cuff tear. The rate of readmission in wheelchair users was greater than in non-wheelchair users (24.05% vs. 9.55%, OR: 3.00, CI: 2.279, 3.946). Patients in the wheelchair cohort exhibited higher rates of complications and comorbidities (*p* < 0.001). Among the most likely to be readmitted after TSA were patients with osteoarthritis, pulmonary heart disease, rheumatoid arthritis, and hypertension (*p* < 0.05). Significant differences in surgical outcomes existed between wheelchair and non-wheelchair users in terms of preoperative comorbidities, postoperative complications, and readmission rates.

## 1. Introduction

In the United States, an estimated 3.3 million individuals use a manual or electric wheelchair as their primary means of mobility, with nearly 2 million of those wheelchair users being over the age of 65 [1,2]. In this wheelchair-dependent population, the prevalence of shoulder pathologies is high, with 33–62% of wheelchair users reporting compromised independent living and decreased quality of life [1,2,3,4].

Previous studies have reported the increased prevalence of shoulder pathologies and overuse of the glenohumeral joint leading to rotator cuff tears and glenohumeral osteoarthritis during wheelchair propulsion and activities such as wheelchair transfer [3,5,6,7,8]. The consensus is that the pathophysiology of weight-bearing shoulders induces glenohumeral joint osteoarthritis via repetitive overuse during wheelchair propulsion. Repetitive overuse causes microtrauma, which can gradually deteriorate the rotator cuff tendons, ultimately resulting in a tear [9]. Subsequently, the mechanical limitations imposed by a rotator cuff tear lead to superior migration of the humeral head, linked to subacromial impingement syndrome [9]. The loss of subacromial space leads to deficiencies within the cartilage, owing to impaired nutritional delivery and further pathoanatomical defects, leading to the progression of glenohumeral osteoarthritis [9].

Despite the increase in the incidence of rotator cuff tears and glenohumeral osteoarthritis as wheelchair users age, the best treatment and management remains controversial [10,11]. Current non-surgical treatment options for this population include physical therapy, corticosteroid injections, orthobiologics, and other over-the-counter medications [10,11]. Repeated intraarticular steroid injections are contraindicated due to their deleterious effects on the soft tissue and only small and transient pain relief, as reported by Mohamadi and colleagues in their meta-analysis [12]. Surgical management of this population includes rotator cuff repair, anatomical total shoulder arthroplasty, and reverse total shoulder arthroplasty. Though rotator cuff repairs were historically believed to be ineffective as a treatment option due to an elevated risk of re-tears and complex tear patterns, a recent study has shown some short-term success in performing primary rotator cuff repairs, with significant improvements in pain, range of motion, and strength in wheelchair users [13]. However, the healing outcomes of primary rotator cuff repairs were significantly lower compared to the healing outcomes in patients who underwent total shoulder arthroplasty [13]. In addition, total shoulder arthroplasty has been reported to minimize pain and improve functional outcomes, with a slightly higher rate of complications compared to primary rotator cuff repairs [4,13,14,15]. Previous studies have revealed a higher prevalence of rotator cuff tears and glenohumeral osteoarthritis among individuals in the wheelchair population compared to non-wheelchair users [16]. Due to the considerable reliance on the upper extremities for independent mobility, comprehensive evidence-based treatment planning is necessary.

Additionally, predicting outcomes is more challenging due to the distinctive biomechanics involved in wheelchair-specific movements, such as propulsion [13]. The relationship between the comorbidity of surgical management and postoperative outcomes is crucial for clinical decision-making regarding wheelchair users, yet it remains poorly understood. The purpose of this study was to assess the 1-year preoperative comorbidities, postoperative complications, and readmission rates after TSA among wheelchair users. We hypothesized that wheelchair use would be associated with increased rates of preoperative comorbidities and postoperative complications within one year of TSA compared to patients who did not use wheelchairs. In addition, we hypothesized that wheelchair users were more likely to be readmitted postoperatively following TSA when compared to non-wheelchair users.

## 2. Materials and Methods

### 2.1. Data Source and Study Design

A retrospective cohort study was conducted using the PearlDiver Patient Records Database (PearlDiver Technologies, Colorado Springs, CO, USA), a nationwide insurance billing database of over 25 million patients. The use of the PearlDiver database allows for large-scale evaluation of comorbidities that are less commonly encountered and, therefore, provides valuable information that otherwise might be overlooked in smaller-scale studies at the institutional level. The records in the PearlDiver Patient Records Database are acquired from Humana’s claims database, de-identified, and released commercially for research purposes. Utilizing the International Classification of Disease, ninth and tenth revision codes, along with the Current Procedural Terminology codes (Table S1), two cohorts of patients—wheelchair users and non-wheelchair users diagnosed with either glenohumeral arthritis or a rotator cuff tear, and who subsequently underwent total shoulder arthroplasty—were created. The 2010 to 2020 Q2 M157Ortho PearlDiver Administrative dataset was utilized to create the two cohorts. These cohorts were matched by age range, gender, and region. Patients were included if their entry in the dataset included the corresponding ICD code batches for rotator cuff tear and total shoulder arthroplasty, which includes both the total shoulder arthroplasty and reverse total shoulder arthroplasty techniques, and the corresponding CPT code for wheelchair use included in our query. Patients were excluded if they did not hold the same insurance coverage for one year prior to and following surgery, ensuring continuity of care and data. This timeframe was selected because it allowed for the greatest amount of transparency in reporting comorbidity and complication data.

### 2.2. Creating the Experimental Cohort

**Figure 1:**
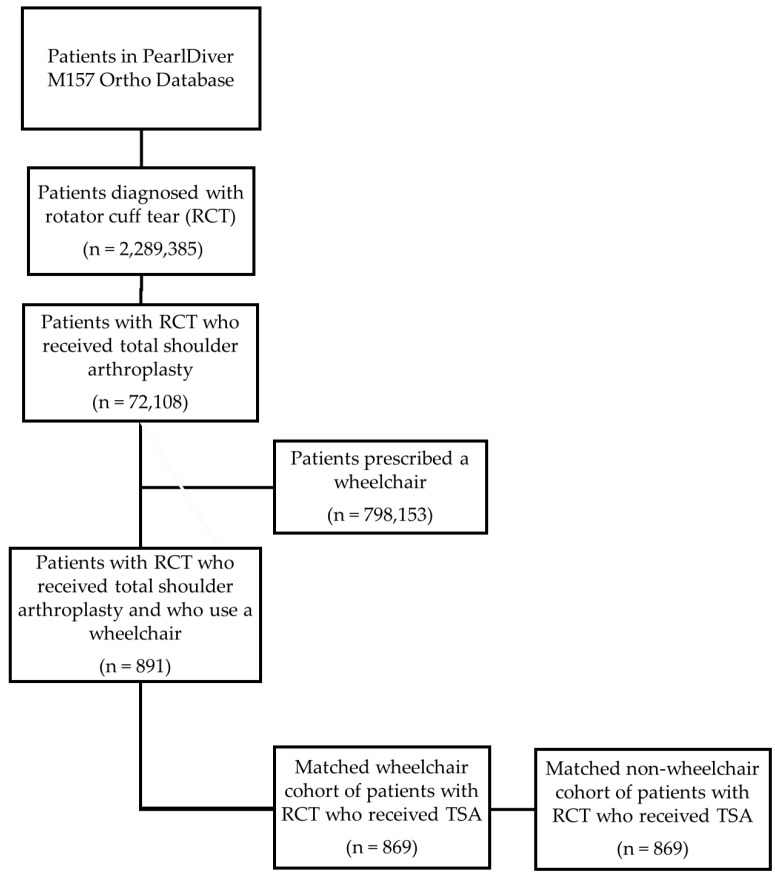
Flowchart outlining the process of creating the two matched cohorts in PearlDiver.

Our initial PearlDiver query identified 2,289,385 patients with an ICD–10 diagnosis of rotator cuff tear. Additionally, we identified 798,193 patients in the database with a CPT or ICD code corresponding to wheelchair use. Subsequently, we combined the two patient populations to identify 72,108 patients who underwent total shoulder arthroplasty with a concurrent diagnosis of rotator cuff tear, among whom 891 patients were also prescribed a wheelchair. Two matched cohorts were created: one with wheelchair users and one with non-wheelchair users, with 869 patients in each cohort, as outlined in Figure 1.

### 2.3. Determining and Comparing the Rates of Readmission, Preoperative Comorbidities, and Postoperative Complications

The PearlDiver database provides a list of the most common preoperative comorbidities and postoperative complications found within its database, as well as the corresponding ICD-9 and ICD-10 codes, and predefines a common variable for each complication and comorbidity within the software for ease of use (Table S1). To ensure the reproducibility of this study, the decision was made to use the predefined PearlDiver list of the most common preoperative comorbidities and postoperative complications. The occurrence of a postoperative complication was determined based on the concomitant presence of a new ICD-9 or ICD-10 code corresponding to the most prevalent post-surgical complications, as defined by PearlDiver, within one year following the procedure. The identification of a preoperative comorbidity was established by the concurrent presence of an ICD-9 or ICD-10 code corresponding to the most prevalent preoperative comorbidities, as defined by PearlDiver, which were pre-existing for a minimum of one year prior to the surgical procedure. Patients were classified as readmissions if they presented to the hospital within 1 year of their surgery with the same active ICD and CPT codes for their visit. One-year medical and surgical outcomes, along with readmission statistics, were extracted and compared between the 2 cohorts. Patients who underwent surgery (TSA) between 2010 and the second quarter of 2020 were analyzed.

For those interested in the complete list of CPT, ICD-9, and ICD-10 codes used in this study, please refer to the Supplementary Materials included with this article.

### 2.4. Statistical Analysis

Univariate analysis was performed to compare the rates of preoperative comorbidities, postoperative complications, and readmission between the wheelchair cohort and the non-wheelchair cohort by calculating odds ratios and 95% confidence intervals (CI). Chi-squared tests were also performed to compare the rates of comorbidities and complications between wheelchair users and non-wheelchair users. A logistic regression was performed to predict readmission rates using the 1-year preoperative comorbidity and postoperative complication rates in wheelchair users. For all statistical analyses, a *p*-value < 0.05 was used to determine statistical significance. All statistical analyses were performed with the embedded R statistical package within PearlDiver.

## 3. Results

### 3.1. Demographic Breakdown of Cohorts

**Figure 2:**
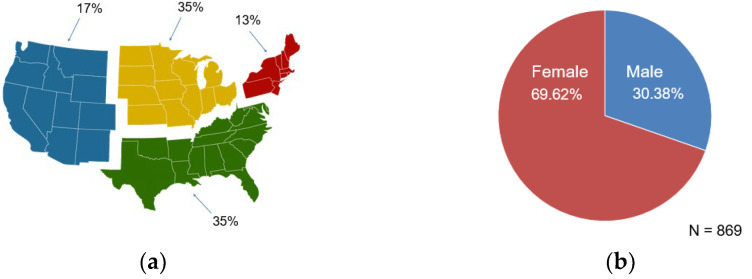
Demographic breakdown of matched cohorts according to (**a**) region and (**b**) gender.

The demographic breakdown of each cohort is detailed in Table 1 and Figure 2.

### 3.2. Readmissions

The results of the logistic regression model demonstrated that the rate of readmission in wheelchair users was greater than that in non-wheelchair users (24.05% vs. 9.55%, OR: 3.00, CI: 2.2788, 3.9462) (Table 2). Wheelchair users were nearly three times more likely to be readmitted following TSA than non-wheelchair users.

In the wheelchair population, patients with osteoarthritis, ischemic heart disease, pulmonary heart disease, chronic kidney disease, and previous arrhythmias were the most likely to be readmitted after TSA (*p* < 0.05) (Table 3).

### 3.3. Postoperative Complications

Medical complications including urinary tract infections (OR: 3.5475, 95% CI: 2.7742, 4.5365), pneumonia (OR: 3.4351, 95% CI: 2.4495, 4.8174), acute kidney injury (OR: 4.5601, 95% CI: 3.2247, 6.4484), deep vein thrombosis (OR: 1.7489, 95% CI: 0.9708, 3.1506), and wound disruption (OR: 2.2154, 95% CI: 1.0784, 4.5511) occurred at greater rates for patients in the wheelchair cohort (*p* < 0.05) (Table 3).

### 3.4. Preoperative Comorbidities

Preoperative comorbidities were found at greater rates in the wheelchair cohort, including previous myocardial infarctions (OR: 2.1060, 95% CI: 1.4781, 3.0005), anemia (OR: 2.6156, 95% CI: 1.5245, 4.4876), arrhythmias (OR: 2.4346, 95% CI: 1.9408, 3.0542), osteoarthritis (OR: 2.0676, 95% CI: 1.7008, 2.5134), rheumatoid arthritis (OR: 1.9333, 95% CI: 1.2369, 3.0217), asthma (OR: 1.8810, 95% CI: 1.4320, 2.4710), hypertension (OR: 2.5611, 95% CI: 2.0399, 3.2153), ischemic heart disease (OR: 3.2646, 95% CI: 1.9774, 5.3897), pulmonary heart disease (OR: 2.6951, 95% CI: 1.8438, 3.9393), obesity (OR: 2.3985, 95% CI: 1.9502, 2.9499), diabetes (OR: 2.4846, 95% CI: 2.0240, 3.0501), chronic obstructive pulmonary disease (COPD) (OR: 2.5983, 95% CI: 2.0600, 3.2773), coronary artery disease (OR: 1.9044, 95% CI: 1.5326, 2.3663), chronic kidney disease (OR: 2.1857, 95% CI: 1.7177, 2.7813), and concurrent history of tobacco use (OR: 1.8599, 95% CI: 1.5179, 2.2790) (*p* < 0.001) (Table 3). However, concurrent opioid use was not found to be significantly different between groups (OR: 0.8381, 95% CI: 0.6723, 1.0448, *p* = 0.0581) (Table 4).

## 4. Discussion

The purposes of this study were to (1) assess the 1-year preoperative comorbidities, postoperative complications, and readmission rates after TSA among wheelchair users and (2) identify risk factors for readmission following total shoulder arthroplasty in wheelchair users. The results of our study support our hypothesis that wheelchair use would be associated with increased rates of preoperative comorbidities and postoperative complications within one year of TSA compared to patients who did not use wheelchairs. Additionally, the results of our study support our hypothesis that wheelchair users would be more likely to be readmitted postoperatively following TSA when compared to non-wheelchair users. Our analysis confirmed that there are significant differences in surgical outcomes between wheelchair and non-wheelchair patients across all three categories: preoperative comorbidities, postoperative complications, and readmission rates.

Our results show that preoperative comorbidities including myocardial infarction, anemia, arrhythmia, osteoarthritis, rheumatoid arthritis, asthma, hypertension, ischemic heart disease, pulmonary heart disease, obesity, diabetes, COPD, coronary artery disease, chronic kidney disease, and tobacco use were more prevalent in wheelchair users than in non-wheelchair users. Previous studies have shown that regular physical activity reduces the risk of developing cardiovascular disease, type 2 diabetes mellitus, obesity, pulmonary disease, kidney disease, and osteoarthritis [17,18,19,20,21,22]. For patients who depend on the use of a wheelchair for mobility, regular physical activity at the level recommended to reduce the risk of developing these comorbidities may be difficult, which could explain the higher prevalence of these comorbidities in the wheelchair population in our study [19]. A previous epidemiological study by Borrelli et al. found that people with mobility impairments had a greater smoking prevalence than people who did not have mobility impairments, which reflects the findings in our study [23].

Additionally, the data demonstrate a greater rate of post-surgical complications in wheelchair users compared to non-wheelchair users, including urinary tract infections, pneumonia, acute kidney injury, deep vein thrombosis, and wound disruption. Previous studies demonstrated that early mobilization in the postoperative period and the ability to walk were associated with decreased rates of urinary tract infection, pneumonia, and deep vein thrombosis [24,25,26]. For wheelchair patients who are unable to walk or are otherwise incapable of this early mobilization, they are at increased risk of urinary tract infections, pneumonia, and deep vein thrombosis [24,25,26]. Postoperative wound disruption has previously been linked to concurrent comorbidities such as diabetes and tobacco use, which inhibit wound perfusion and healing [27]. These same comorbidities were found in greater rates in the wheelchair population in our study.

Our study’s results showed that wheelchair users were nearly three times more likely to be readmitted than non-wheelchair users within the first year following TSA, with readmission rates of 24.05% vs. 9.55%, respectively. Previous studies have found similar 90-day readmission rates for non-wheelchair users (anywhere between 5.9% and 7.3%) following TSA [28,29]. Our analysis further demonstrates that comorbidities such as osteoarthritis, ischemic heart disease, pulmonary heart disease, chronic kidney disease, and previous arrhythmias are also strong predictors of readmission. Several risk factors associated with readmissions following total shoulder arthroplasty in wheelchair users were comparable with non-wheelchair users [13,15,21]. Dislocation and infection are the leading causes for readmission following TSA [30]. However, our results differed with respect to urinary tract infections, acute kidney injury, and would disruption as the leading causes of readmission in our wheelchair user cohort. These differences are likely due to the considerable limitations in independent mobility and ambulation postoperatively and are otherwise common medical challenges in patients with a spinal cord injury [31,32].

The implications of this study can guide patient counseling and shape the discussion of postoperative expectations between surgeons and patients in this unique but growing population. Understanding that wheelchair users are at greater risk of certain complications, present with greater rates of comorbidities, and are more likely to be readmitted can prompt preventative care decisions such as postoperative antibiotic therapy, long-term symptom and functional monitoring, and planning for physical therapy and rehabilitation.

This study utilized a large insurance database to assess the surgical outcomes of treating shoulder pathologies in wheelchair users. Conversely, conducting a comparable study utilizing data from a single institution and obtaining meaningful findings with broad applicability would have required extensive time and resources. The relatively uncommon patient population of wheelchair users would likely lead to small sample sizes and insufficient power for single institutional studies. The use of a large database also minimizes differences in study outcomes due to patient demographics, especially when assessing potential differences in surgical outcomes between geographical regions in the US.

While one of the advantages of this study is the ability to analyze the extracted clinical data from a diverse patient population across the entire country, there are several limitations as pertains to using a large administrative database such as PearlDiver. One of the constraints of using PearlDiver is the inability to account for subgroups with a small number of patients, due to the de-identified nature of the database. This limitation arises during attempts to break down cohorts further for sub-analysis. This can happen based on stratification of cohorts by demographic categories such as the patient’s age, ethnicity, or region of the US. This occurrence can also arise when stratifying cohorts based on comorbidities or complications. PearlDiver will not return the population of a subgroup if there are fewer than 10 patients in the subgroup, so as to protect patient privacy. Due to the privacy restrictions, the preoperative and postoperative periods used for the analysis had to be extended to one year to minimize sample size insufficiency and increase the statistical power for the analysis of surgical outcomes and readmission statistics in the wheelchair population. Additionally, we were unable to isolate any specific confounding variables through univariate or multivariate analysis. One measure taken to minimize the effects of confounding pre-existing comorbidities was to selectively include only patients with an active diagnosis of that comorbidity in the one-year period preceding TSA repair. By selecting a one-year threshold, it is possible that we excluded a significant number of patients from our cohorts. Furthermore, this form of analysis, which is dependent on the accuracy of medical billing coding, is inherently at risk for miscoding due to human error. However, previous studies have shown that the use of a large administrative dataset with a large study sample size and a large patient population in each cohort should mitigate the effects of miscoding during analysis [33]. A limitation of relying only on ICD-9 and -10 and CPT codes to establish the wheelchair cohort is the inability to further analyze the specific use patterns of wheelchair users. Breaking down the cohort by variables such as the total time using a wheelchair each day, type of wheelchair (mechanical or electric), or the duration of wheelchair use since first use was not possible.

Future prospective longitudinal studies are needed to elucidate complications, readmission rates, and the risk of revision surgery for wheelchair users following total shoulder arthroplasty. The present study identified key variables to be addressed in future cohort studies. Additionally, efforts to further stratify the wheelchair usage patterns within another nationwide database, such as the National Spinal Cord Model System database, will shed more light on confounding variables. Further research on the efficacy of non-surgical treatment options and overall outcomes compared to surgery could also influence the decision-making process between surgeons and patients dependent on wheelchairs for mobility.

## 5. Conclusions

The results of this study demonstrate that wheelchair users are at risk of a higher complication profile following TSA compared to non-wheelchair users. Therefore, the findings suggest that patients should be adequately counseled on the risks and benefits before electing to pursue surgical interventions. Specifically, an important consideration during pre-surgical counseling is adequately identifying the optimal length of immobilization and rehabilitation during the postoperative period. Such individualized care would aid in clear communication between the surgical team, rehabilitation team, patient, and the patient’s family, therefore conceivably minimizing postoperative complications.

## Figures and Tables

**Table 1:** Demographic breakdown of matched cohorts by age; an asterisk (*) indicates that the information in that category has been blinded due to insufficient sample volume.

Age Range (Years)	Number of Patients in Each Age Range	Percentage of Patients in Each Age Range
35–39	*	*
40–44	*	*
45–49	11	1.27%
50–54	28	3.22%
55–59	74	8.52%
60–64	127	14.61%
65–69	159	18.30%
70–74	194	22.32%
75–79	211	24.28%
80+	56	6.44%
Mean age + SD: 75.1 ± 4.1		

**Table 2:** Rates of readmission between wheelchair users and non-wheelchair users undergoing TSA.

	Wheelchair Users (*n* = 869)		Non-Wheelchair Users (*n* = 869)		Odds Ratio	95% CI	*p*-Value
Readmissions following TSA	209	24.05%	83	9.55%	2.9988	2.2788, 3.9462	<0.0001

**Table 3:** Predictors of preoperative comorbidities for 1-year postoperative readmission in wheelchair users undergoing arthroplasty; * statistically significant *p* < 0.05.

Comorbidity	Readmission Within 1 Year (*n* = 209)		No Readmission (*n* = 660)		*p*-Value
Osteoarthritis	79	37.80%	296	44.85%	0.0367 *
Rheumatoid arthritis	17	8.13%	34	5.15%	0.0565
Asthma	36	17.22%	114	17.27%	0.4936
Hypertension	171	81.82%	530	80.30%	0.3144
Ischemic heart disease	16	7.66%	38	5.76%	0.0114 *
Pulmonary heart disease	35	16.75%	52	7.88%	0.0002 *
Obesity	83	39.71%	251	38.03%	0.3315
Diabetes	92	44.02%	272	41.21%	0.2367
Coronary artery disease	67	32.06%	202	30.61%	0.3462
Chronic kidney disease	60	28.71%	150	22.73%	0.0392 *
Tobacco use	69	33.01%	243	36.82%	0.1589
Myocardial infarction	26	12.44%	61	9.24%	0.0898
Anemia	11	5.26%	26	3.94%	0.2044
Arrhythmia	76	36.36%	192	29.09%	0.0236 *

**Table 4:** Comparison of complication and comorbidity rates between wheelchair users and non-wheelchair users; * statistically significant *p* < 0.05.

Arthroplasty	Wheelchair Users (*n* = 869)		Non-Wheelchair Users (*n* = 869)		OR	95% CI	*p*-Value
Postoperative complications							
Urinary tract infection	291	33.49%	108	12.43%	3.5475	2.7742, 4.5365	<0.0001 *
Pneumonia	148	17.03%	49	5.64%	3.4351	2.4495, 4.8174	<0.0001 *
Acute kidney injury	170	19.56%	44	5.06%	4.5601	3.2247, 6.4484	<0.0001 *
Deep vein thrombosis	31	3.57%	18	2.07%	1.7489	0.9708, 3.1506	0.0180 *
Wound disruption	24	2.76%	11	1.27%	2.2154	1.0784, 4.5511	0.0076 *
Preoperative comorbidities							
Myocardial infarction	99	11.39%	50	5.75%	2.1060	1.4781, 3.0005	<0.0001 *
Anemia	48	5.52%	19	2.19%	2.6156	1.5245, 4.4876	0.0001 *
Arrhythmia	288	33.14%	147	16.92%	2.4346	1.9408, 3.0542	<0.0001 *
Opioid use	646	74.34%	674	77.56%	0.8381	0.6723, 1.0448	0.0581
Osteoarthritis	425	48.91%	275	31.65%	2.0676	1.7008, 2.5134	<0.0001 *
Rheumatoid arthritis	58	6.67%	31	3.57%	1.9333	1.2369, 3.0217	0.0009 *
Asthma	163	18.76%	95	10.93%	1.8810	1.4320, 2.4710	<0.0001 *
Hypertension	725	83.43%	576	66.28%	2.5611	2.0399, 3.2153	<0.0001 *
Ischemic heart disease	65	7.48%	21	2.42%	3.2646	1.9774, 5.3897	<0.0001 *
Pulmonary heart disease	100	11.51%	40	4.60%	2.6951	1.8438, 3.9393	<0.0001 *
Obesity	367	42.23%	203	23.36%	2.3985	1.9502, 2.9499	<0.0001 *
Diabetes	384	44.19%	210	24.17%	2.4846	2.0240, 3.0501	<0.0001 *
COPD	281	32.34%	135	15.54%	2.5983	2.0600, 3.2773	<0.0001 *
Coronary artery disease	286	32.91%	178	20.48%	1.9044	1.5326, 2.3663	<0.0001 *
Chronic kidney disease	235	27.04%	126	14.50%	2.1857	1.7177, 2.7813	<0.0001 *
Tobacco use	346	39.82%	228	26.24%	1.8599	1.5179, 2.2790	<0.0001 *

## Data Availability

All data underlying this article will be shared upon reasonable request to the corresponding authors.

## Supplementary Materials

The following supporting information can be downloaded at: https://www.mdpi.com/article/10.3390/jcm12185799/s1, Table S1: List of all ICD and CPT codes used.
