# Supplementary material for: Perioperative Outcomes, Comorbidities, and Complications following Total Shoulder Arthroplasty in Wheelchair Users: A Retrospective Cohort Analysis of a Nationwide Database
Source: J Clin Med. 2023 Sep 6;12(18):5799. doi: 10.3390/jcm12185799 (PMC10532280; doi:10.3390/jcm12185799)
Supplement: Supplementary file 1 [file jcm-12-05799-s001.zip › jcm-2553221-supplementary.pdf]

Table S1: List of All CPT and ICD Codes Used

CPT/ICD Defintion	Corresponding Code
<u>CPT Codes</u>	
Total Shoulder Arthroplasty	CPT-23472
Reverese Total Shoulder Arthroplasty	CPT-23472
Wheelchair Users	CPT-97542
<u>ICD-9 and ICD-10 Codes for Diagnoses</u>	
Rotator Cuff Tear	ICD-10-D-M75100, ICD-10-D-M75101, ICD-10-D-M75102, ICD-10-D-M75110, ICD-10-D-M75111, ICD-10-D-M75112, ICD-10-D-M75120, ICD-10-D-M75121, ICD-10-D-M75122
Glenohumeral Osteoarthritis	ICD-10-M19
Wheelchair Users	ICD-10-D-Z993
<u>ICD-9 and ICD-10 Codes for Comorbidities</u>	
Osteoarthritis	ICD-9-D-71500:ICD-9-D-71599,ICD-10-D-M1911:ICD-10-D-M1993
Rheumatoid Arthritis	ICD-9-D-7140, ICD-9-D-7142, ICD-10-M0520:ICD-10-D-M061
Asthma	ICD-9-D-49300, ICD-9-D-49399, ICD-10-D-J452:ICD-10-D-J45988
Hypertension	ICD-9-D-4010:ICD-9-D-4059, ICD-10-D-I10:ICD-10-D-I159

Ischemic Heart Disease	ICD-9-D-41000:ICD-9-D-41499, ICD-10-D-I21:ICD-10-D-I229
Pulmonary Heart Disease	ICD-9-D-4150:ICD-9-D-41799, ICD-10-D-I26:ICD-10-D- I279
Obesity	ICD-9-D-2780, ICD-9-D-27800, ICD-9-D-27801, ICD-9-D-27802, ICD-9-D-27803, ICD-10-D-E660:ICD-10-D-E669
Diabetes	ICD-9-D-24900:ICD-9-D-25099, ICD-9-D-7902, ICD-9-D-79021, ICD-9-D-79022, ICD-9-D-79029, ICD-9-D-7915, ICD-9-D-7916, ICD-10-D-E090:ICD-10-D-E139
Chronic Obstructive Pulmonary Disease	ICD-9-D-490:ICD-9-D-49699, ICD-10-D-J44:ICD-10-DJ449
Coronary Artery Disease	ICD-9-D-4110:ICD-9-D-4149, ICD-10-D-I25:ICD-10-D-I259
Congestive Heart Failure	ICD-9-D-39891, ICD-9-D-4280, ICD-9-D-4281, ICD-9-D-42820, ICD-9-D-42821, ICD-9-D-42822, ICD-9-D-42823, ICD-9-D-42830, ICD-9-D-42831, ICD-9-D-42832, ICD-9-D-42833, ICD-9-D-42840, ICD-9-D-42841, ICD-9-D-42842, ICD-9-D-42843, ICD-9-D-4289, ICD-10-D-I150:ICD-10-D-I159
Chronic Kidney Disease	ICD-9-D-585, ICD-9-D-5851, ICD-9-D-5852, ICD-9-D-5853, ICD-9-D-5854, ICD-9-D-5855, ICD-9-D-5856, ICD-9-D-5859, ICD-9-D-7925, ICD-10-D-N18:ICD-10-D-N189
Tobacco Use	ICD-9-D-3051, ICD-9-D-V1582, ICD-10-D-F17220, ICD-10-D-F17221, ICD-10-D-F17223, ICD-10-D-F17228, ICD-10-D-F17229, ICD-10-D-F17290, ICD-10-D-

	F17291, ICD-10-D-F17293, ICD-10-D-F17298, ICD-10-D-F17299, ICD-10-D-Z720
<u>ICD-9 and ICD-10 Codes for Complications</u>	
Acute Kidney Injury	ICD-9-D-5845, ICD-9-D-5846, ICD-9-D-5847, ICD-9-D-5848, ICD-9-D-5849, ICD-10-D-N17:ICD-10-D-N179
Urinary Tract Infection	ICD-9-D-5990, ICD-10-D-N390
Pneumonia	ICD-9-D-4800:ICD-9-D-4809, ICD-9-D-481, ICD-9-D-4820, ICD-9-D-4821, ICD-9-D-48230, ICD-9-D-48231, ICD-9-D-48232, ICD-9-D-48239, ICD-9-D-48240, ICD-9-D-48241, ICD-9-D-48242, ICD-9-D-48249, ICD-9-D-48281, ICD-9-D-48282, ICD-9-D-48283, ICD-9-D-48284, ICD-9-D-48289, ICD-9-D-4829, ICD-9-D-4830, ICD-9-D-4831, ICD-9-D-4838, ICD-9-D-4841, ICD-9-D-4843, ICD-9-D-4845, ICD-9-D-4846, ICD-9-D-4847, ICD-9-D-4848, ICD-9-D-485, ICD-9-D-486, ICD-10-D-J12:ICD-10-D-J189
Deep Vein Thrombosis	ICD-9-D-4532, ICD-9-D-4533, ICD-9-D-4534, ICD-9-D-45382, ICD-9-D-45384, ICD-9-D-45385, ICD-9-D-45386, ICD-10-D-I26:ICD-10-D-I2699
Wound Disruption	ICD-9-D-99830, ICD-9-D-99831, ICD-9-D-99832, ICD-9-D- 99833, ICD-10-D-T8130XA, ICD-10-D-T8130XD, ICD-10-D-T8130XS, ICD-10-D-T8131XA, ICD-10-D-T8131XD, ICD-10-D-T8131XS, ICD-10-D-T8132XA, ICD-10-D-T8132XD, ICD-10-D-T8132XS, ICD-10-D-T8133XA, ICD-10-D-T8133XD, ICD-10-D-T8133XS
